# Emergency Braking Intention Detect System Based on K-Order Propagation Number Algorithm: A Network Perspective

**DOI:** 10.3390/brainsci11111424

**Published:** 2021-10-27

**Authors:** Yuhong Zhang, Yuan Liao, Yudi Zhang, Liya Huang

**Affiliations:** 1 College of Automation and College of Artificial Intelligence, Nanjing University of Posts and Telecommunications, Nanjing 210023, China; b18040421@njupt.edu.cn (Y.Z.); b18040334@njupt.edu.cn (Y.L.); b18041109@njupt.edu.cn (Y.Z.); 2 College of Electronic and Optical Engineering and College of Microelectronics, Nanjing University of Posts and Telecommunications, Nanjing 210023, China; 3 National and Local Joint Engineering Laboratory of RF Integration and Micro-Assembly Technology, Nanjing 210003, China

**Keywords:** brain-computer interface technology (BCI), electroencephalogram (EEG), braking intention detect, brain network, K-order structure entropy, pattern recognition

## Abstract

In order to avoid erroneous braking responses when vehicle drivers are faced with a stressful setting, a *K-order propagation number algorithm–Feature selection–Classification System (KFCS)* is developed in this paper to detect emergency braking intentions in simulated driving scenarios using electroencephalography (EEG) signals. Two approaches are employed in KFCS to extract EEG features and to improve classification performance: the K-Order Propagation Number Algorithm is the former, calculating the node importance from the perspective of brain networks as a novel approach; the latter uses a set of feature extraction algorithms to adjust the thresholds. Working with the data collected from seven subjects, the highest classification accuracy of a single trial can reach over 90%, with an overall accuracy of 83%. Furthermore, this paper attempts to investigate the mechanisms of brain activeness under two scenarios by using a topography technique at the sensor-data level. The results suggest that the active regions at two states is different, which leaves further exploration for future investigations.

## 1. Introduction

Approximately 3700 traffic participants are killed by road accidents in the world everyday [1]. Among all of the potential causes, mistakes are commonly made once human beings are in stressed physical settings. For instance, a driver could step on the accelerator incorrectly when an emergency happens in front of the moving-fast vehicle when his original intention was to hit the braking pedal. This erroneous action can cause severe consequences such as lethal injuries or even mortalities.

Research studies have presented that it is feasible to identify emergency braking intentions by investigating driver’s Electroencephalogram (EEG). For example, Haufe et al. [2] asked subjects to maneuver simulated driving equipment and drew the conclusion that the EEG signal at emergency cases can be detected earlier than muscular actions; the former is about 130 ms faster than the latter, and this leading duration is equivalent to 3.5 m reduced at the speed of 100 kmph.

The prevailing detection methods generally focus on EEG’s frequency-domain and time-domain characters. The Fourier families, for example, Discrete-time Fourier transform (DFT) and Discrete Wavelet Transformation (DWT), are commonly used. Teng et al. [3] analyzed five frequency bands, and sequential forward floating searches (SFFS) were deployed as a fresh approach to discover the optimal feature sets. As a result, the accuracy of detection can be greater than 90%. Nonetheless, the experiment paradigm they designed took only one special scenario into account, which applied a sudden crossing as the emergency state when the automobile moved at the speed of 108 kmph.

Similarly, Nguyen and Chung [4] studied five bands’ power spectra by using FFT at frequency-domain and used auto-regressive (AR) to process time sequences at the time-domain, which resulted in 91% accuracy and 600 ms outstripping under emergency scenarios. However, it is regrettable that their driving scenarios are insipid as well: missing blended data between urban and rural area and nighttime and daytime. On the other hand, with the exception of studying braking movements on straight roads, Guo et al. [5] also explored scenarios on curved roads. Moreover, Independent components analysis (ICA) and power spectral density (PSD) were acquired to pre-treat and process EEG signals separately.

In addition to capturing EEG features with traditional time and frequency perspectives, a brain network angle is on the rise as well [6]. This shows that our brain is made up of hundreds of billions of neurons for which their electrical interactions formed a complex network [7]. Generally, there are two categorized approaches to model this giant, i.e., structural and functional brain networks. The former is related to an anatomical field by using techniques such as fMRI, and the latter is measured by nodes’ connection defined by communications within electrical electrodes [8]. Meanwhile, three steps are necessary to construct the functional model: (1) defining network nodes and edges; (2) computing the connectivity between edges to generate adjacency matrix; and (3) computing complex network parameters. Each step is important for gaining results [9]. For the adjacency matrix, there are two forms, which are weighted and unweighted. In the former, any diagonal elements in the matrix are nonzero, while the latter can also refer to 0–1, i.e., binarized network.

Currently, a number of studies using a brain network to capture EEG features have been carried out. Firstly, functional brain network methods are trending in the field of treating neurological diseases, for instance, Ahmadi et al. [10] used functional network parameters to classify epilepsy and psychogenic non-epileptic seizures subjects, and Fang et al. [11] investigated glioma-related epilepsy patients by using network parameters as well. Secondly, beyond neuroscience, the practice of network methods can be observed in other fields such as educational assessment and cognitive studies. Specifically, Chang et al. [12] recognized strangers and acquaintance based on brain network parameters, and 90% accuracy rate in the delta band was realized. Wang et al. [13] designed a lie testing experiment and mainly focused on parameters that can characterize small-world properties, which is considered to better model neural systems [14,15,16,17]. Thirdly, although a weighted brain network can preserve inclusive information about the connectivity between electrodes, it significantly increases the complexity of calculating EEG features, and it may contain a large amount of information redundancy, which is the so called low signal-to-noise ratio [18]. Thus, finding an appropriate threshold value to binarize the weighted brain network is another important issue in the task of classifying EEG signals [19,20,21,22]. Nonetheless, some studies discussed threshold value selection strategies for binarizing weighted networks, while most did not. Ahmadi et al. [23]’s approach paralleled former researchers in that both time-domain and brain network features were treated as input to classify alcoholism; however, the process of choosing threshold values was empirical. Ai et al. [24] used a feature fusion scheme, including network parameter features, to classify four patterns in a motor imaginary trial. However, the procedures of choosing the threshold values and the applications of the brain network in the field of safety driving are generally lacking.

Therefore, in order to improve the imperfections of simulated driving environments in previous studies, this paper will use a more dynamic, true-to-life and comprehensive simulating driving scenario. Additionally, to fill out the gap in the field of using brain network features to classify EEG signals, this paper applies brain network features for EEG braking signal classification, and an original method, i.e., K-Order Propagation Number Algorithm, proposed by our team is used to extract network features [25,26]. This method can measure the heterogeneity and node importance of networks under various network scales, i.e., it not only considers its local nodes but also takes into account remote ones. Then, the features are sent to a Support Vector Machine (SVM) in order to obtain results. The highest classification accuracy rate can be achieved above 90% with the most appropriate threshold value, and the overall accuracy rate is around 83%.

This paper is arranged as follows: Section 2 introduces the experiment details. Section 3 explicates the classification of system architecture and the methods that are used in this paper, particularly the K-Order Propagation Number Algorithm. Section 4 demonstrates the results and analysis. Conclusions and discussions are listed in Section 5.

## 2. Experiment

Due to the uneasiness of designing experiments and obtaining EEG data in authentic driving settings, e.g., our EEG cap and amplifier are rather unwieldy than compared to handy ones in obtaining effective EEG signals, we are driven to test the validity and performance of K-Order Propagation Number Algorithm in primarily simulated driving scenarios. Once the feasibility of the algorithm has been proven, then we can further devise more sophisticated experiment schemes using more portable equipment in real settings. Hence, we first explicate our experiment equipment.

### 2.1. Experiment Equipment: Software and Hardware

A driving simulation game developed by SCS Software Inc. (Prague, Czech Republic), named European Truck Simulator 2, was used as the driving simulation software in the experiment. Unlike previous studies that only have a few essential road elements such as pedestrians and crossing vehicles, in this simulation software, subjects need to drive a truck to complete the task of cargo transportation. Subjects can select more than 60 cities as the starting location and several transportation routes that are linked with it. The driving setting generally includes two scenarios: city roads and highways. In each scenario, there will be situations such as randomly generated speed limit, traffic lights, day or night time backgrounds, sunny to rainy days and so on, which considerably increased the veracity of the driving experience.

**Figure 1:**
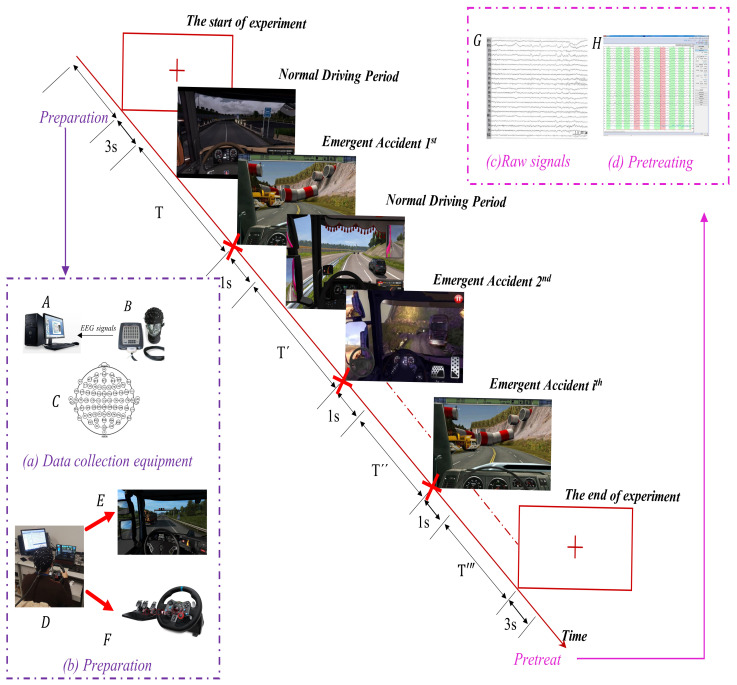
Experiment paradigm. The reddish diagonal line represents a comprehensive work flow of one experiment, including firstly the preparation blocks: (**a**) data collection equipment and (**b**) experiment preparation. Then, the formal experiment begins, and the subject drive at two states, i.e., braking and normal driving. When the experiment ends, technicians download (**c**) raw EEG signal data and employ algorithms to (**d**) pre-treat the data for subsequent use.

The driving simulator uses a Logitech G29 driving suite, which includes a steering wheel and pedal device. The EEG signal acquisition equipment is the 64-channel signal amplifier, matched EEG cap and Curry software, version 7.0 developed by Compumedics Neuroscan (Charlotte, NC, USA) (Figure 1).

### 2.2. Experiment Paradigm

At the beginning of the experiment, the subjects focused on a reddish cross on a screen and retained it for 3 s, as shown in Figure 1. Next, they began to start a truck to perform formal driving from the starting location, usually a warehouse. Then, the subjects drove to the destination indicated by a map.

When the vehicle passed the urban road, traffic flow was intensive, and red traffic lights occurred frequently; thus, the subjects had high levels of vigilance. After entering the highways, however, due to the long route and fewer signal lights, the subjects’ vigilance level decreased, and they would feel fatigued after a long period of driving, which increased the possibility of false responses when an emergency event happened.

The emergency braking actions happened at two main circumstances: red lights in front of the vehicle at crossroads and overtaking vehicles appearing from behind. When the subject performed emergency braking, the experiment assistant artificially marked it as a braking event, and this EEG segment lasted for 1 s. The interval between two emergency incidences is the normal driving period ***T***; the experiment assistant also needs to take 1 s signal sample from this period as a parallel to the braking EEG samples. This normal-braking-normal cycle repeated to ensure one trial can obtain copious samples of normal and braking states.

Nonetheless, given that the completion of a cargo transportation task would sometimes last more than 40 min due to the software program settings, this could add heavy mental burdens to the subject. For the sake of subjects’ focus ability, once a single simulation driving trial exceed 40 min, the experiment assistant records that trial as a completed task. However, if one cargo transportation task lasts far less than 30 min, say 10 or 20 min, the subject has to restart another task until his or her driving duration reached at least 30 min. Then, the subject looked at the reddish cross on the screen for 3 s again, and one trial is completed.

### 2.3. Experiment Preparation

Before the formal start of the experiment, the experiment assistant stated detailed experiment procedures to the subjects and asked them whether they agree with the content. Based on their willingness, they decided whether to sign the informed consent sheet or not. Each subject in our experiment is assured that they agreed with the full terms listed on the informed consent sheet.

To be specific, subjects will understand the gist of the preparation and formal experiment procedures they are involved in. These main points are listed as follows: (1) All subjects are required to concentrate on the driving task completely, and all scenes as well as emergency events will occur arbitrarily, which strengthens the validity of the final results. (2) After the subjects are completely prepared, the experiment assistants will put on the EEG cap and prepare the conductive paste for gluing in order to guarantee the resistance of 64 channels that are low enough to collect serviceable EEG signals. (3) To become familiar with the driving environment and equipment, subjects needed to maneuver the simulation software twice before the formal trial. However, to avoid extra tiredness, this adaptation has to be compelted at least 20 min earlier than the formal driving task.

After subjects understood and signed the informed consent sheet, the experiment assistants will begin to help subjects to execute the preparation procedure as mentioned above.

### 2.4. Subject Information Outline

We invited four male and three female subjects to participate in the experiments (with an age range from 19 to 23; the average age is 21). All subjects are equipped with skills in operating a vehicle with manual or automatic clutch and are in good health and have no ophthalmic deficiency. They all have right-handed preferences.

## 3. Methods

### 3.1. KFCS Architecture

**Figure 2:**
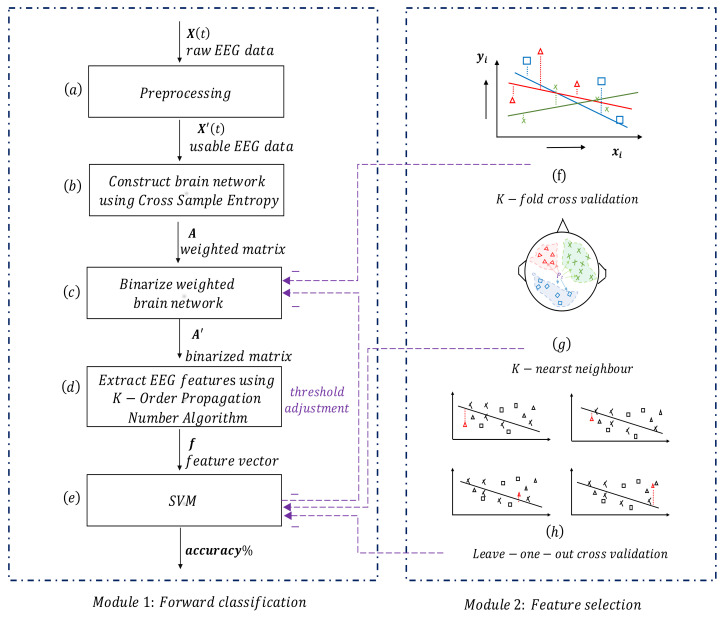
KFCS architecture: two modules. The first one is the *Forward classification module*, which is the main module. The second *Feature selection module* adjusts the threshold value in order to improve classification results in the first module. (**a**) Signal preprocessing block, which is used to preprocess raw EEG signals. (**b**) Cross-Sample Entropy (CsEn) algorithm is used to construct brain network. Block (**c**) is used to binarize weighted network obtained by CsEn. (**d**) K-Order Propagation Number Algorithm is employed to extract brain network features. (**e**) Support Vector Machine algorithm is the classifier. (**f**) K-fold Cross Validation method is used to calculate the overall classification accuracy and select the appropriate threshold. (**g**) K-nearest Neighbor (KNN) algorithm and (**h**) Leave-one-out Cross Validation (LOOCV) algorithm are used to do feature selection. X(t) is raw EEG data sequences generated from different sensor channels; $X^{\prime }\left(t\right)$ usable EEG data after preprocessing; A is weighted network adjacency matrix calculated by using CsEn algorithm; $A^{\prime }$ is binarized (0–1) matrix; f is the feature vector computed by using K-Order Propagation Number Algorithm; accuracy% is the classification accuracy rate measured in percentile.

An original *K-order propagation number algorithm–Feature selection–Classification System (KFCS)* is constructed to detect emergency braking intentions, and it is divided into two modules, as shown in Figure 2.

The first module is used to forward classify pretreated EEG signals, that is, generating SVM results using node importance features. The general descriptions of blocks in the first module are enumerated as follows. (a) The signal preprocessing block is used to pre-process raw EEG signals to obtain classifiable data. (b) This block is used to construct a brain network by using Cross-Sample Entropy (CsEn) algorithm, which is employed to compute the connectivity of each pairwise electrodes in order to obtain adjacency matrix *A* of the brain network. (c) Given that *A* is a weighted network, this block uses adaptive threshold values that are determined by traversing to binarize *A*. (d) The K-Order Propagation Number Algorithm block is a novel method proposed by our team and is used to extract features of brain networks. (e) The SVM classification algorithm block.

The second module’s function is to select threshold values with respect to different subjects that could produce the best classification results: (f) The K-fold Cross Validation method is used to calculate the overall classification accuracy at each threshold, and the result is used to select the appropriate binarization threshold. (g) The K-nearest Neighbor (KNN) algorithm. (h) The Leave-one-out Cross Validation (LOOCV) algorithm.

For the sake of clarification, the first forward module has to be run at least once before the second module plays its part, and two modules do not work in parallel; instead, the second one is functions more similarly to a sub-module relative to the first one, and it helps the first module in increasing the total classification accuracy rate.

### 3.2. Raw EEG Data Preprocessing

Given the fact that the strength of the EEG signal is very weak that its magnitude fluctuation range will normally not exceed tens of microvolts, which is easily submerged by various noises and artifacts [27], the purpose of preprocessing is, therefore, to (a) eliminate artifacts, which consist of unrecognizable segment caused by muscle movement. Moreover, Independent Component Analysis (ICA) is used here [28] to (b) cancel out the frequency component, including unusually high and low frequency bands. Normally, the frequency range of noise is considered to be within the range of 50–60 Hz; in our experiment, 0.5–45 Hz is considered to be the effective range of EEG signals.

### 3.3. Construct Brain Network Using Cross Sample Entropy

Currently, there are many effective signal processing approaches for converting physiological micro electrical activity into local cortical connections, for instance, Phase locking value (PLV) [29], Granger Causality, Cross Approximate Entropy (CsApEn) and Cross Sample Entropy (CsEn) [30].

CsEn is employed in this paper to calculate the quantity of connection between two electrodes because of its good noise cancellation performance and relatively high accuracy results with respect to relatively less EEG data.

The CsEn is defined as follows:

(1) $$Cross\_SampleEntropy\left(\pi ,r\right)=\frac{-ln(W^{m}\left(dt\right)(x\left(t\right)||y\left(t\right)))}{Z^{m}\left(dt\right)\left(x\left(t\right)||y\left(t\right)\right)}$$

where π is the feature dimension, *r* is the distance threshold, x(t) and y(t) are different time sequences, dt is the given distance threshold and $W^{m}$ and $Z^{m}$ are two reference parameters. The formula of CsEn determines that the brain network generated by each sample sequence is undirected and weighted; however, in order to reduce computational complexity, the weighted connectivity network has to be binarized.

### 3.4. Binarize Weighted Brain Network

After obtaining connective measuring matrix, a threshold value $Tr_{i}$ is introduced to binarize the matrix in order to obtain an unweighted and undirected brain network; the sparsity of the network is largely affected by $Tr_{i}$: the greater the value, the stricter it is with respect to forming a connection between two channels mathematically and vice versa. The ranges of different $Tr_{i}$ under various subjects are determined by the min-max values in the CsEn matrices; then, step size is settled based on the tradeoff between low computational complexity and better result accuracy.

Each subject’s brake and non-brake classification accuracies under different threshold $Tr_{i}$ are given by SVM, and the highest accuracy with its corresponding $Tr_{i}$ can be obtained by ranking. However, various subjects’ accuracy rate and corresponding $Tr_{i}$ are of great inconsistency; therefore, extra work had to be performed in order to guarantee robust methods, that is, the feature selection module is about to play its part and will be provided in detail in Section 3.6. Before coming into Section 3.6, however, as introduced above, the forward classification module has to function independently at least once; thus, the next section introduces a newly developed approach to extract features from a network perspective.

### 3.5. Extract EEG Features Using K-Order Propagation Number Algorithm

The K-Order Propagation Number Algorithm is a novel method proposed by our team, and it is used to compute the node importance feature of a complex network. One remark, however, is that the K-order Structure Entropy that was first introduced by Huang et al. [25] and then Tang et al. [26] was combined with Weighted Ranking algorithms in order to derive this K-Order Propagation Number Algorithm. The inspiration of this algorithm is from the problem of modeling infectious disease propagation, which is mentioned as follows.

Modelling the propagation of infectious disease is a classic problem, but it is still of great pragmatic significance. Every outbreak of infectious disease has profound impacts on human society, such as the transmission of cholera in London in 1849 and the COVID-19 global pandemic [31,32]. Various methods have been employed to model the mechanism of infectious diseases propagation, such as classical SIR model and its improved versions based on coupled differential equations; statistical model and computational simulation approaches utilizing the power of modern computers and vast amount of data distribution; and network perspective models based on node-edge topological analysis, which is the main focus in this paper [33].

Considering the individual differences of propagation ability in the transmission model of infectious diseases and simultaneously viewing each infected biological entity, say a person, as a node in the network and this person’s propagation ability as the step size *K*, i.e., the population can be infected by that patient in one unit time, then each node’s relative importance is obtained by calculating information entropy of all *K*s, with each node’s step size *K* varying. Consequently, when combining all nodes’ relative importance, the heterogeneity of the network can be measured. Intuitively, if all nodes’ importance parameters are have low variance distribution, then there is no eminent node in the network; as a result, we cannot say that this network is of great heterogeneity and vice versa.

Compared with other methods that measure the heterogeneity of networks, such as DD entropy and Wu entropy, this method has apparent superior advantages in that it can more precisely describe the communication characteristics of a network [25]. Moreover, when using a deliberate attack scheme to destroy a network, the *K-Order Propagation Number Algorithm* can make a network at a low communication efficiency state operate faster than other approaches [26]. However, as mentioned before, the *K-Order Propagation Number Algorithm* can only be operated under unweighted and undirected networks.

The steps to derive K-Order Propagation Number Algorithm are explained as follows:

(1) Computing brain network adjacency matrix: Let each channel be a node $v_{i}$. Based on the connectivity parameter $e_{ij}$ between two channels calculated in the previous section, i.e., CsEn, the weighted network G(V,E) is given, where $V=\{v_{1},v_{2},v_{3},\ldots ,v_{64}\}$, 64 nodes in total. In $E=\{e_{11},e_{12},\ldots ,e_{ij}\}$, $e_{ij}$ denotes the edge between $v_{i}$ and $v_{j}$. *A* is the weighted adjacency matrix of the network.
(2) Defining the *K-order neighbourhood number*: In the brain network, the number of nodes that a particular node $v_{i}$ can reach under the step size *K* is $N_{v_{i}}^{K}$ and $N_{v_{i}}^{K}=\sum_{i=1}^{n}I\left(l_{ij}\leq K\right)+1$, where $l_{ij}$ denotes the shortest path between two nodes $v_{i}$ and $v_{j}$; n=64 in this paper. When $l_{ij}\leq K$, the indication function is I(·)=0; otherwise, it is I(·)=1. With the propagation step size *K*, if the number of nodes that $v_{i}$ can reach is higher than $v_{j}$, then we have reason to believe that the influence of $v_{i}$ is greater than $v_{j}$, i.e., $v_{i}$ is more important.
(3) Derivation of the *K-Order Structure Entropy* formula: By combining the K-order neighborhood number $N_{v_{i}}^{K}$ with the information entropy formula, the *K-Order Structure Entropy* formula can be derived as follows:
  (2) $$H^{K}=-\sum_{i=1}^{n}\frac{N_{v_{i}}^{K}}{\sum_{j=1}^{n}N_{v_{j}}^{K}}log\left(\frac{N_{v_{i}}^{K}}{\sum_{j=1}^{n}N_{v_{j}}^{K}}\right),K\in \left\{0,1,\ldots ,d\right\}$$
  where *d* is the diameter of the network, i.e., the number of nodes that comprises the longest route in the network.
(4) Derivation of *K-Order Propagation Number Algorithm*: The entropy values $H^{K}$ of node $v_{i}$ under all step sizes *K* are comprehensively computed; then, they are normalized and weighted in order to obtain the node importance parameter $Q_{v_{i}}$ of $v_{i}$:
  (3) $$Q_{v_{i}}=\sum_{K=0}^{d}c^{K}\cdot S_{v_{i}}^{K}$$
  where $S_{v_{i}}^{K}$ is the normalized result of $N_{v_{i}}^{K}$, and $c^{K}$ is the weighted coefficient, which employs a mathematical treatment to focus more on *K* moments when there exist great node importance differences and can downplay the specific *K* moment when the node importance difference is small. It is defined by the following:
  (4) $$S_{v_{i}}^{K}=\frac{N_{v_{i}}^{K}-\min\left(N^{K}\right)}{\max\left(N^{K}\right)-\min\left(N^{K}\right)},N^{K}=\left\{N_{{}^{v_{1}}}^{K},N_{{}^{v_{2}}}^{K},\cdots ,N_{{}^{v_{n}}}^{K}\right\}$$
  and the following.
  (5) $$c^{K}=1-\frac{H^{K}-\min\left(H\right)}{\max(H)-\min(H)},H=\left\{H^{0},H^{1},\cdot \cdot \cdot ,H^{d}\right\}$$
(5) The feature vector *f* is evaluated by sorting all nodes’ importance parameters $Q=\{Q_{v_{1}},Q_{v_{2}},\ldots ,Q_{v_{n}}\}$, n=64. Repeat the above steps for the brain network’s adjacency matrix at each sample moment (brake or non-brake instance), and then all the feature vectors $f_{1\times 64}$ of one trial (a 30–40 min simulated driving) can be calculated.

### 3.6. Feature Selection Module

To improve KFCS’s robustness, that is, we hope to find a threshold value that can ensure that all subjects can have relatively high classification accuracy rate, in this paper, this value is termed as the *General Optimal Threshold Value (GOTV)*. After the first-round of operations by the forward classification module, the *10-fold cross-validation method* [34] is used to compute this GOTV. As a result, the threshold value adaptive to one subject can be easily found based on GOTV. Therefore, the classification time could be greatly saved for developing a personalized brake intention detection system.

Meanwhile, by considering 64 channels as nodes in our paper, they are of higher feature dimensions, and with small sample sets, a larger feature dimension could worsen the result [35]. Thus, the number of nodes that is used to extract features should be reduced as much as possible but without sacrificing high classification rate.

K-nearest Neighbor (KNN) and Leave-one-out Cross Validation LOOCV are employed here to select features. The main idea of KNN is that each feature can be represented by its k-nearest neighborhood feature, i.e., grouping a series of regional nodes as a feature set $M_{i}$, i∈{1,2,3,…,64} [36]. Then, the core node $v_{c}$ is selected from different feature sets by using LOOCV [37]. Thus, in the latter rounds of SVM classification, these core nodes can substitute the original 64 nodes.

## 4. Result Analysis

In this experiment, approximately 400 braking samples were collected through all subjects, i.e., 400 one-second EEG braking signal segments and another 400 normal driving samples as counterpart.

### 4.1. CsEn Analysis

**Figure 3:**
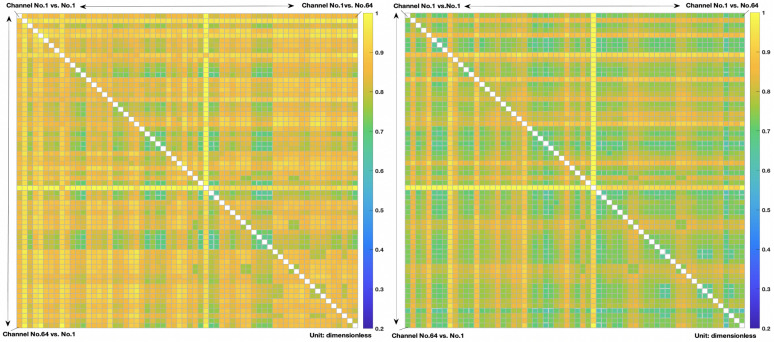
Two CsEn adjacency matrices generated by a group of sampled data with mean CsEn value after normalization under two driving states. CsEn matrix diagram of sampled group data at normal driving condition is shown on the left hand side; CsEn matrix diagram of sampled group data at braking condition is shown on the right hand side. The greater the CsEn value, the lesser the correlation degree between two time series and vice versa [30]. The mean CsEn value of this group of sampled data at braking states is lower than the normal driving state, which indicates that brain activities are generally more intense at braking states in these two samples.

By analyzing all normalized CsEn adjacency matrices of all subjects corresponding with two driving states, a great difference can be observed in the matrix diagrams; that is, the entropy value during the braking state is lower than the normal driving state. Since the lower the entropy value, the stronger the correlation between two channels [30], this means that, generally, the brain activity in the particular regions where the subject was performing a braking action is stronger. We extracted nearly 50% CsEn samples of each of the seven subjects at two driving states and normalized the values of each matrix point after averaging them; the CsEn matrices of the two states at group level are shown in Figure 3. After normalization, the mean values of CsEn matrix at two states are 0.8395 in the normal driving state and 0.7819 during the braking condition, respectively.

**Figure 4:**
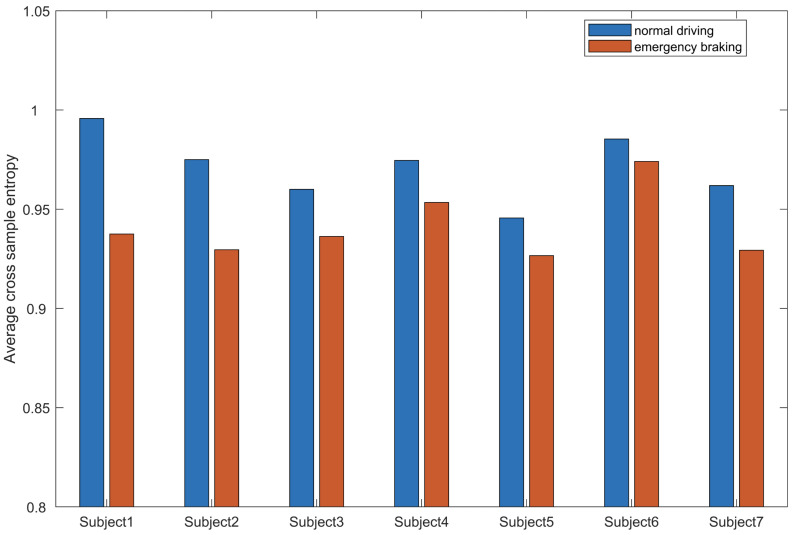
Seven subjects’ brain network mean CsEn values between braking and normal driving states. The overall mean CsEn value of all 7 subjects at normal driving states is greater than at braking states; the values for normal driving and braking state are 0.975273 and 0.933746, respectively. Moreover, each subject’s general mean CsEn value at normal states was greater than at braking states as well, which shows good consistency.

The mean CsEn values of all seven subjects under two driving states are shown in Figure 4. The CsEn values in normal driving condition are greater than those in the emergency braking condition as a whole. The overall mean CsEn value of normal driving state is 0.975273, and for emergency braking states, it is 0.933746.

### 4.2. Node Importance Feature and Explanation

**Figure 5:**
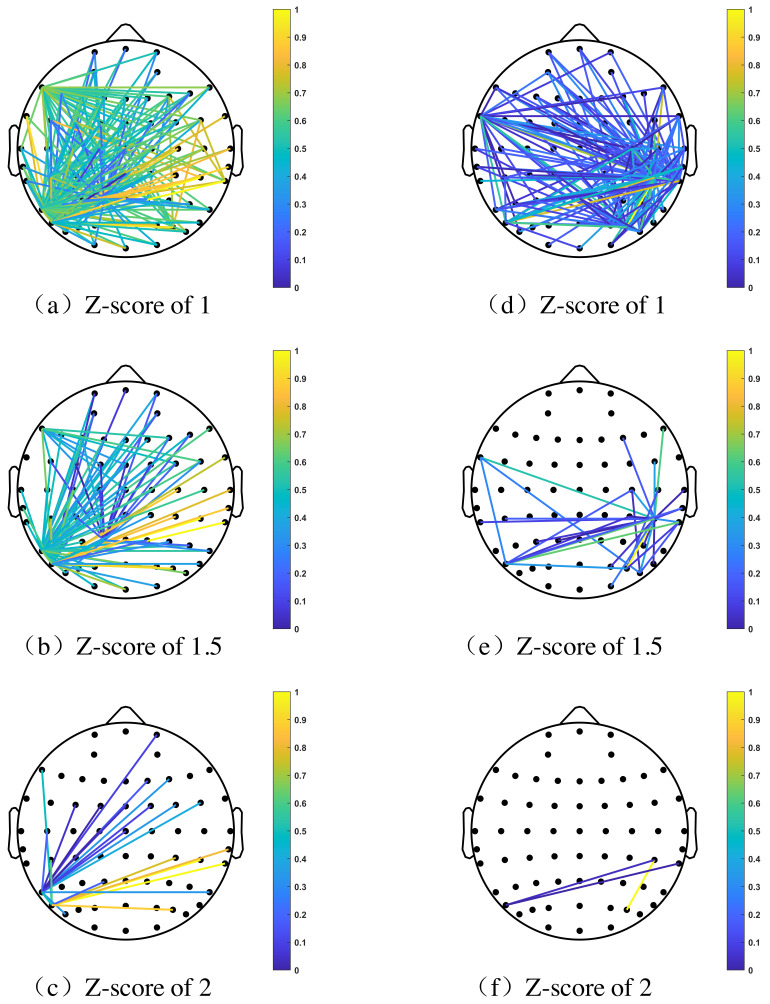
Brain network connectivity density of subject No.6 changed with the variation of threshold during normal driving state (**a**–**c**), and the density changed with the variation of threshold during the braking state (**d**–**f**).

Similarly, by generating topography graphs of brain connectivity density network of all EEG samples under various threshold values, a notable difference is displayed between two driving states. We randomly chose two EEG samples with respect to each driving state of subject No.6, as shown in Figure 5 for comparison. We used Z-score to measure the deviation of the threshold value from the average level. At each state, brain network connections corresponding to 3 Z-scores were presented, which are Z=1, Z=1.5 and Z=2. When Z-score varies from a small value to a larger one, the threshold value increases from the small level to a high level accordingly. Therefore, as shown in the Figure 5, under a small threshold (a small Z-score), there are more edges in the brain network. At larger thresholds (larger Z-score), the network becomes sparser, reflecting where the brain is active at a given state (at the sensor-level).

For subject No.6, the most intensively connected brain nodes during braking states were generally located in the right occipital lobe region, an area known to be associated with vision, image recognition and perception [38]. The brain nodes with the highest connectivity during periods of normal driving were generally positioned at the junction between the left occipital lobe and the left parietal lobe regions. The function of the left occipital lobe is thought to be related to muscle and skin sensation, while most unconscious movements can be attributed to parietal lobe regions [38].

A possible explanation can be used to explain the mechanism of uneven distribution of the most intensive connection nodes. Body movements controlled by the brain are mainly divided into unconscious and conscious. Unconscious movements are those acts that do not require much awareness; most of acts in our daily life are unconscious, such as blinking. On the other hand, movements requiring ’high-level’ brain involvement are categorized as conscious, for example, taking water from a cup requires central nerves system to receive and send signals. Moreover, conscious acts can transform into unconscious acts by massive training [39].

Therefore, when the subject is at a normal driving state, several conscious acts such as holding the acceleration peddle transformed into unconscious acts, and parietal lobe regions are responsible for it. However, in both driving conditions, emergency braking acts particularly required more visual attentions; as a result, the left occipital lobe region of the subject is somehow active.

### 4.3. Classification Result Analysis

The mean GOTV of seven subjects is achieved at 0.75; the highest was 0.89 for subject No.6, and the lowest was 0.68 for subject No.5. Each subject’ optimal threshold value fell within an interval centered at GOTV with a standard deviation of 0.073, as observed in Table 1.

**Figure 6:**
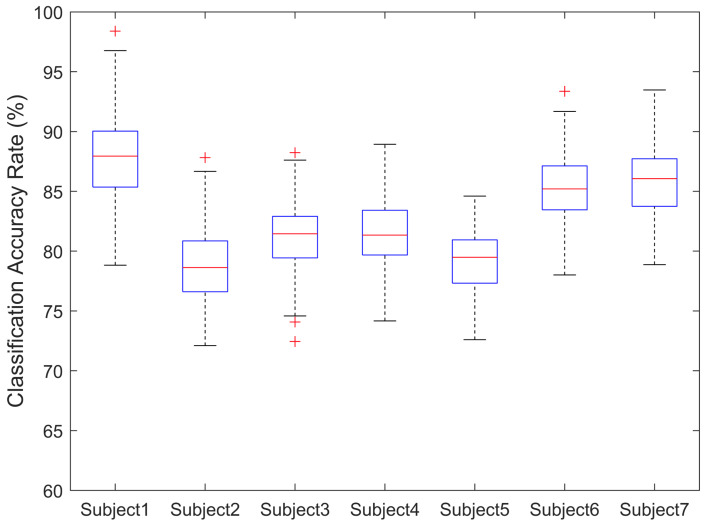
Box plot of 7 subjects’ classification accuracy rate. The overall classification accuracy results of 7 subjects are largely distributed within the approximate range from 75% to 90%. The highest overall mean classification accuracy was 87% achieved by subject No.1; the mean classification accuracy of 7 subjects was about 83%. Each ‘+’ means one outlier result of this group.

The highest classification results of SVM are achieved after the improvement of feature selection, and they are shown in Figure 6. The mean classification accuracy of seven subjects was 83%, and the highest mean classification accuracy was 87% in subject No.1. Meanwhile, the best classification performance of a single trial can be achieved at 100%, which is also by subject No.1. After feature selection, the classification accuracy of each subject was improved to a greater extent, with a relative accuracy improvement rate of 19.85% in subject No.1 and an overall average improvement rate of 7.83% for all other subjects.

## 5. Conclusions and Discussion

In this paper, EEG data of seven subjects in a simulation driving experiment were used to construct a CsEn brain network, and a novel method proposed by our research team, K-Order Propagation Number Algorithm, was used to analyze the features of the brain network under two states, i.e., braking and non-braking, and also to detect drivers’ intention during emergency braking. Meanwhile, a series of feature selection schemes are employed to (1) work out a General Optimal Threshold Value (GOTV); and (2) to improve classification results by reducing feature dimension. All of the EEG signal processing blocks and feature selection algorithms constitute a robust system, KFCS.

In comparison with current research, several virtues of this paper are highlighted as follows: (1) In addition to time-domain and frequency-domain approaches, we employed an entropy-based method to extract EEG feature; that is, the EEG features of subjects are calculated from a novel network perspective. This method assists practitioners in gaining insights of brain mechanisms intuitively, especially in helping to determine the activeness of brain regions under specific circumstances. (2) A comprehensive procedure to calculate threshold values of subjects’ feature networks was demonstrated as a paradigm for future practitioners to refer to. (3) Moreover, it is worth mentioning again that the threshold values have been calculated twice before obtaining final results. For the first time, a GOTV is computed, which is significant not only as being constructive in this paper but it can also be beneficial for achieving an average threshold level when there is a group of subjects who are waiting in line to obtain their own threshold value in other experiments. Based on GOTV, a set of adaptive thresholds with regard to specific subjects can then be quickly obtained; this two-step procedure can save a great deal of time if there is a real-time system requirement. (4) The simulated driving experiment scenarios in this paper are more dynamic and more realistic in terms of mimicking real-world driving situations, vehicles, landscape background and weather conditions, etc. Moreover, it can provide drivers with a fantastic driving experience, which could in turn validate the results.

However, there are some limitations in this work that need to be considered in subsequent studies. First, since we used simulation driving equipment to collect EEG data, many potential factors could deteriorate the results, such as fluctuations generated by vehicles on uneven paved roads and freer body movements differing from laboratory conditions than those that are mandatory in a real car situation, are beyond the scope of this paper. Second, the features that are chosen as SVM’s inputs are not sufficiently wide-ranging. Only EEG features that are extracted from the brain network are used as input to classifier, but multimodal data or MultiModal Machine Learning (MMML) methods can actually be more effective when portraying the target digital event; in this case, we neglected to use EMG and facial recognition data to improve classification accuracy [40,41]. Third, the real-time performances have not been studied well enough. On the one hand, the time that is needed to process the entire KFCS is not available. On the other hand, laboratory equipment such as the 64-channel brain cap and signal amplifiers are unwieldy, which cannot be easily transfered from location to location. Fourth, the qualification of subjects should be further determined [42]. In this paper, the subjects were selected randomly from a narrow range, i.e., mainly from our university. However, diverse age ranges and skillfulness in driving are also effective with respect to the results since the activeness of brain regions can be reasonably different during conscious and unconscious body movements. Fifth, another problem was the connectivity that we measured, which was at sensor-level. Due to the volume conduction (field spread) effect, there will always be some spurious-free interactions between different brain regions; thus, any attempt to investigate brain connection mechanisms will be somewhat biased [43,44].

Our future investigations will address the following issues: (1) Real car settings ought to be used to design and carry out the experiment, including various road conditions, speed and other traffic signal indicators; (2) extracting a wider range of digital data, including but not limited to visual data, EMG and Eye movement signal, etc., to construct a multimodal data platform. Meanwhile, we will carry out EEG frequency dynamic analysis when the brain performs certain tasks. (3) We hope to design and test an integrated embedded system with high real-time performance and a system that is highly adaptive in order to minimize emergency braking detection time for drivers from a wide range class. (4) We also wish to design a more stable and portable EEG signal acquisition device with fewer channels in the future so that we can perform our experiment in real-time settings. (5) Finally, we hope to solve the problem of volume conduction and since our data has good spatial resolution, we will try to measure connectivity at the source level so that we can better explore the mystery of the most important organ of human body, the brain, according to Schoffelen and Gross [44]. 

## Figures and Tables

**Table 1:** GOTV of 7 subjects and each subject’s optimal threshold value.

Subject No.	1	2	3	4	5	6	7
Optimal Threshold Value	0.75	0.69	0.79	0.7	0.68	0.89	0.75
Mean	0.75						
Standard Deviation	0.073						
